# Determinants of community-acquired pneumonia among under-five children in Awi Zone, Northwest Ethiopia

**DOI:** 10.3389/fpubh.2025.1511263

**Published:** 2025-05-01

**Authors:** Nigussie Adam Birhan, Alene Yirsaw Workineh, Zelalem Meraf Wolde, Emebiet Abich, Gedif Mulat Alemayehu, Atalaye Nigussie, Yenew Alemu, Chalachew Alemie Messfin, Denekew Bitew Belay

**Affiliations:** ^1^Department of Statistics, College of Natural and Computational Science, Injibara University, Injibara, Ethiopia; ^2^Injibara General Hospital, Injibara, Ethiopia; ^3^One Acre Fund, Bahir Dar, Ethiopia; ^4^Department of Statistics, College of Science, Bahir Dar University, Bahir Dar, Ethiopia; ^5^Department of Statistics, University of Pretoria, Pretoria, South Africa

**Keywords:** Awi Zone, community acquired pneumonia, determinants, upper respiratory tract infection, under-five children

## Abstract

**Background:**

Globally, community-acquired pneumonia is the leading cause of death in under-five children, accounting for 7.6 million deaths. Among these deaths, approximately 99% occur in low and middle-income countries. The present study aimed to assess the magnitude of community-acquired pneumonia and its associated factors among under-five children in Awi Zone.

**Methods:**

A community cross-sectional study was conducted on 1,368 participants from March to July 2023. A multistage sampling method was used. Data were entered into Epi-Data and exported to STATA for analysis. Bivariable and multivariable logistic regressions were used. Variables with a *p*-value of < 0.05 were considered statistically significant.

**Results:**

The percentage of community-acquired pneumonia among under-five children was 11.33% (95% CI: 9.75–13.12%). Primary education [AOR = 0.38; 95% CI:0.15, 0.95], stunting [AOR = 4.80; 95% CI: 2.31, 9.94], diarrhea [AOR = 3.75; 95% CI: 1.96, 7.18], acute lower respiratory tract infection [AOR = 14.57, 95% CI: 3.18, 66.78], acute upper respiratory tract infection [AOR = 9.06; 95% CI: 2.03, 40.42], and presence of separate kitchen [AOR = 0.38, 95% CI: 0.20, 0.72] were associated with community-acquired pneumonia.

**Conclusion:**

In this study, the percentage of community-acquired pneumonia was relatively high. Hence, we recommend adequate health education in areas such as nutritional intervention, the prevention and early treatment of diarrhea and acute respiratory tract infections (ARTI), and preventing indoor air pollution to reduce the risk of community-acquired pneumonia.

## Introduction

Community-acquired childhood pneumonia is an infection of the lower respiratory tract that affects the lungs (1). Pneumonia is an inflammation of the lungs, involving the bronchioles and the functional unit of the lung (2). It can be categorized as either non-infectious, including conditions such as aspiration, resulting from inhalation of food, gastric contents, or other foreign material, or infectious, including causes such as bacteria, viruses, fungi, or parasites (3). Specific symptoms such as cough with sputum production, fever, chest pain, shortness of breath, and chills are the main characteristics associated with pneumonia (4). The precise definition and diagnosis of pneumonia are still up for debate for a number of reasons, including the difficulty in determining the etiological agents in individuals, the low specificity of lower respiratory tract infection symptoms, and the limited accessibility of laboratory tests and imaging (5).

The presence of cough and fast breathing and/or difficulty breathing, based on age-specific criteria, confirms the classification of suspected pneumonia in under-five children (6, 7). According to the World Health Organization (WHO), fast breathing has been defined as a respiratory rate of >60/min for infants less than 2 months age; >50/min for infants of 2–12 months age, and >40/min for children more than 12–59 months of age (7).

Across the world, pneumonia affects approximately 156 million under-five children every year. Among these, approximately 151 million live in developing nations (8). In Sub-Saharan Africa, approximately 4 million cases of pneumonia occur annually, resulting in approximately 200,000 deaths (9). Globally, according to the WHO, approximately 7.6 million under-five children of age die annually due to pneumonia (10). Among these deaths, more than 99% were in low-and middle-income countries (11).

In Ethiopia, pneumonia is a leading cause of death among under-five children (12). It is estimated that 3,370,000 children encounter pneumonia annually, which contributes to 20% of all causes of death, which can be easily prevented and treated through simple and cost-effective interventions (13).

Previous studies indicated that some of the most common risk factors for pneumonia include lack of exclusive breastfeeding, living in a crowded house, using charcoal for cooking, using previous upper respiratory tract infection, more than four family members, absence of a separate kitchen, absence of a window in the kitchen, age of the child, history of diarrhea in the child, and stunting (14–18).

Even if a high number of prevention strategies are performed in the Awi Zone prevent pneumonia, the disease remains a public health problem. Controlling the ongoing threat of pneumonia is one of the major health priorities of the Federal Ministry of Health (FMoH) of Ethiopia, to which this study aimed to contribute its part. Although several studies have examined the risk factors for pneumonia at broader regional and national levels (8, 19, 20), there is a lack of specific research focusing on the study area. The risk factors may vary based on local sociodemographic, environmental, and healthcare access conditions; understanding these factors in the study area is essential for designing targeted interventions. The outcomes of the present study have the potential to benefit various stakeholders, including policymakers, organizations working on pneumonia and its risk factors, and future researchers. This information can be used as additional evidence for planning and implementing intervention strategies to prevent CAP and reduce the burden on the community. The contributing factors need to be studied to better inform and educate policy makers, programmers, implementers, and the general population about the problem. Therefore, this study aimed to assess the magnitude and identify risk factors of community-acquired pneumonia among under-five children in Awi Zone, Northwest Ethiopia.

## Materials and methods

### Study design, setting, and period

This study was conducted in Awi Zone, which is one of the 10 zones in the Amhara region of Ethiopia. According to the Central Statistical Agency, 2007, the estimated total population of 982,942, of whom 491,865 were men and 491,077 were women (21). It is bordered on the west by the Benishangul-Gumuz region, on the north by the West Gondar zone, and on the east by the West Gojjam zone. It is located within 114 km from Bahir Dar, the capital of the Amhara region, and 449 km from Addis Ababa, which is the capital city of Ethiopia.

Awi Zone elevations vary from 1,800 to 3,100 m above sea level, with an average altitude of approximately 2,300 m. It has a total of 15 districts, 6 urban and 9 rural, 5 governmental hospitals, 46 health centers, 125 private clinics, and 1 hospital that provides preventive and curative services to the community (22). A community-based cross-sectional study design was used from March to June 2023.

### Study and source population

The source population consisted of all mothers who had under-five children living in the households of Awi Zone, while the study population included all mothers who had under-five children pair living in randomly selected kebeles in the Awi Zone at the time of study.

### Inclusion and exclusion criteria

Mothers/caregivers with child pairs living in Awi Zone at least for 6 months prior to data collection were incorporated into this study. However, mothers/caregivers with child pairs having resided in the research area for less than 6 months prior to data collection, as well as those who did not volunteer to participate, mothers/caregivers who were critically ill, or seriously ill during data collection, were excluded from this study.

### Sample size determination and sampling technique

Sample size was computed based on single population proportion formula using 3% margin of error. The prevalence of pneumonia among under-five children in Munesa District, Arsi Zone, Oromia Region, Ethiopia is 17.7% (23), and the sample size was calculated as follows at 95% confidence level:


$$n=\frac{p\left(1-p\right){Z_{\alpha /2}}^{2}}{d^{2}}=\frac{0.177\left(1-0.177\right)1.96^{2}}{0.03^{2}}=621.79$$

We consider a design effect of 2 as we planned to use a multi-stage cluster sampling technique (Awi Zone-districts-kebeles-households) and a 10% non-response rate (24). The final sample size was 621.79×2 = 1243.58, and by adding the 10% non-response rate, the final sample size was 1,368.

The household numbers having under-five children were taken from health extension workers’ registration books. The youngest child was selected from a household having two or more under-five children, and one child was randomly chosen from among twin births. A multistage sampling technique was used to select study subjects among the 15 districts, which were stratified into urban and rural. Eight districts (three urban and five rural districts) were selected by the lottery method from a clustered sample frame as the primary sampling unit. Similarly, 6 urban and 29 rural kebeles from each urban and rural district were chosen using a simple random sampling technique in the second stage. The first household was selected randomly at the center of the kebeles, and the subsequent households were selected systematically. The sampling interval used for selected kebeles was calculated by dividing the total number of households in each kebele to the allocated sample size. Finally, a total of 1,368 households were selected with probability proportional to population size using a systematic random sampling technique. Mothers who had under-five children were interviewed to obtain information about the history of under-five children in the 5 years preceding the surveys.

### Operational/standard definitions

Community-acquired pneumonia: An acute infection of less than 14 days’ duration, acquired in the community, affecting the lower respiratory tract, with cough and difficulty in breathing, along with fast breathing and/or fever and chest in drawing (14).

Household history of acute lower respiratory tract infection (ALRTI): A household with a history of pneumonia or bronchitis in the last 15 days prior to data collection (25).

Household history of acute upper respiratory tract infection (AURTI): A child whose family has a history of ear infection, common cold, tonsillitis, or pharyngitis in the last 15 days prior to data collection (25).

### Data collection technique and quality control

The data were collected using a face-to-face interviewer-administered questionnaire that was adapted from different works of literature reviewed, including variables such as socioeconomic and demographic, nutritional status characteristics, common childhood illnesses and related care practices, and home-based characteristics.

The data were collected by eight data collectors and supervised by two experienced supervisors with a degree in public health officers and nurses. During the collection period, all questionnaires were reviewed, supervised, and followed up by the supervisors and principal investigators, and then, the necessary feedback was provided to data collectors before the next procedures begin. The original questionnaires were prepared in English, translated into Amharic and Agewegna (local languages), and then translated back to English to check their consistency.

The quality of the data was assured by a properly designed and pre-tested questionnaire, proper training of the interviewers and supervisors, and proper categorization and coding of the data collection instrument before conducting the study. The questionnaire was pre-tested on 5% of study participants in kebeles not included in the main survey before the actual survey, and necessary modifications were conducted.

### Variables in the study

The outcome variable for this study was the occurrence of community-acquired pneumonia, reported by the mother/caregivers of the child and coded as “Yes = 1” and “No = 0.”

In this study, socioeconomic and demographic variables including place of residence, education status of the mother, occupational status of mother, household wealth index, age of the child, sex of the child, mothers’ marital status, number of children less than 59 months in HH, family size, current age of mother, birth order, media exposure, and religion, nutritional status characteristic variables including stunting, duration of breast feeding status, and age at complementary feeding, common childhood illnesses and related care practice variables including vaccination status, history of lower respiratory tract infection in the last 2 weeks, history of upper respiratory tract infection in the last 2 weeks, having diarrhea in the last 2 weeks, and place of delivery, and home-based characteristic variables including toilet facility for HH, source of drinking water for HH, cooking practices in the family, child stays during cooking, and presence of separate kitchen for household were included.

### Data management and method of analysis

During and at the end of data collection, all questionnaires were checked for completeness. The data were entered and cleaned in Epi Info version 4.0.2 and analyzed using STATA 14. The descriptive statistics were conducted to determine the prevalence of community-acquired pneumonia, and analyses were presented using frequency tables and percentages.

Bivariable and multivariable logistic regression models were used to analyze the associations between community-acquired pneumonia and independent variables. In the bivariable logistic regression analysis, the crude odds ratio (COR) was applied to identify factors associated with CAP. All variables with a *p*-value of <0.25 in the bivariable analysis were chosen for the multivariable logistic regression analysis to compensate for confounders (26). In the multivariable analysis, the adjusted odds ratio (27) was used to determine factors associated with community-acquired pneumonia in under-five children, which were expressed at a 95% confidence interval.

## Results

### Percentage, signs, and symptoms of pneumonia

**Figure 1:**
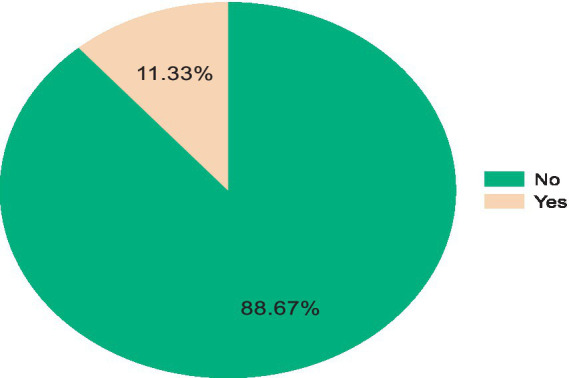
Percentage of community-acquired pneumonia among under-five children in the Awi Zone.

A total of 155 (11.33%) (95% CI: 9.75–13.12%) children had experienced community-acquired pneumonia in the 2 weeks preceding the study (Figure 1).

Of the participating children, 174 (12.72%) had a cough, 155 (11.33%) had fast breathing, 283 (20.69%) had fever, and 160 (11.70%) of the children had indrawn chest (Table 1).

**Table 1:** Percentage of signs and symptoms of community-acquired pneumonia in the Awi Zone, Northwest Ethiopia, 2023.

Characteristics	Categories	Frequency	Percentage
Children who had a cough	No	1,194	87.28
	Yes	174	12.72
Children who had fast breathing	No	1,213	88.68
	Yes	155	11.33
Children who had fever	No	1,085	79.31
	Yes	283	20.69
Children who had in drawn chest	No	1,208	88.30
	Yes	160	11.70

### Home-based characteristics of respondents

Among the study participants, 1,034 (75.58%) have a separate kitchen for cooking, only 19 (1.32%) have electricity for cooking, and 899 (65.72%) children stay on their mothers’ backs while cooking. Concerning toilet availability, 1,309 (95.69%) of the households have a toilet facility (Table 2).

**Table 2:** Home-based characteristics for predictors of community-acquired pneumonia among under-five children in the Awi Zone, Northwest Ethiopia, 2023.

Characteristics	Categories	Frequency	Percentage
Cooking practices in the family	Wood	1,060	77.49
	Charcoal	290	21.20
	Electricity	18	1.32
Whether child stays during cooking	Out of the cooking room	457	33.41
	On mother back	911	66.59
Presence of separate kitchen for household	No	334	24.42
	Yes	1,034	75.58
Main source of drinking water	Unprotected	225	16.45
	Protected	1,143	83.55
Toilet availability of family	No	100	7.31
	Yes	1,268	92.69

### Nutritional characteristics among under-five children

Among the total participants, approximately 939 (68.64%) children breastfed for greater than 12 months and 354 (25.88%) were stunted, whereas 1,014 (74.12%) of them were normal (Table 3).

**Table 3:** Nutritional status characteristics of predictors of community-acquired pneumonia in under-five children in the Awi Zone, Northwest Ethiopia, 2023.

Characteristics	Categories	Frequency	Percentage
Duration of breastfeeding status	12 months and less	429	31.36
	Greater than 12 months	939	68.64
Nutritional status of the child’s height for age (41)	Normal	1,014	74.12
	Stunting	354	25.88
Beginning of complementary feeding at 6 months	Not starting	116	8.48
	No	541	39.55
	Yes	711	51.97

### Common childhood illnesses and related care practices

According to this study, approximately 1,086 (79.39%) children were fully vaccinated, and 234 (17.11%) had no history of diarrhea (Table 4).

**Table 4:** Common childhood illnesses and related care practices as predictors of community-acquired pneumonia among under-five children in the Awi Zone, Northwest Ethiopia, 2023.

Characteristics	Categories	Frequency	Percentage
History of lower respiratory tract infection in the last 2 weeks	No	146	10.67
	Yes	1,222	89.33
History of upper respiratory tract infection in the last 2 weeks	No	1,212	88.60
	Yes	156	10.40
Child vaccination status	Not fully	282	20.61
	Fully	1,086	79.39
History of having diarrhea in the last 2 weeks	No	1,134	82.89
	Yes	234	17.11
Place of delivery	Home	90	6.58
	Health institution	1,278	93.42

### Results of the sociodemographic characteristics of respondents

From a total of 1,368 study participants, 468 (34.21%) of the respondents were living in a rural area. Regarding the family wealth index, approximately 43% of the respondents were categorized as poor. The majority of respondents, 1,300 (95.03%), were married. A total of 299 (21.86%) had attained secondary education and higher, and 725 (53.00%) had no formal education. A majority of the respondents, 766 (55.99%), have family sizes of five and above. The most commonly reported occupation was farming, involving 761 respondents (55.63%) (Table 5).

**Table 5:** Sociodemographic characteristics of households as predictors of community-acquired pneumonia among under-five children in the Awi Zone, Northwest Ethiopia, 2023.

Characteristics	Categories	Frequency	Percentage
Residence	Urban	468	34.21
	Rural	900	65.79
Sex of child	Male	771	56.36
	Female	597	43.64
Religion	Orthodox	1,287	94.08
	Other	81	5.92
Educational status of the mother	No education	725	53.00
	Primary	344	25.15
	Secondary and above	299	21.86
Marital status	Married	1,300	95.03
	Other^*a^	68	4.97
Main occupation of mothers	Farming	761	55.63
	Government employed	77	5.63
	Housewife	450	32.89
	Other^*b^	80	5.85
Age of mother	24 and below	124	9.06
	25–34	736	53.80
	35 and above	508	37.13
Child birth order	1	332	24.27
	2–3	506	36.99
	4–5	324	23.68
	6 and above	206	15.06
Family size of the household	less than 5	602	44.01
	5 and above	766	55.99
Number of children in the family	one	1,035	75.66
	2 and above	333	24.34
Age of the child in months	6 and less than	121	8.85
	7–11	206	15.06
	12–23	382	27.92
	24–59	659	48.17
Wealth index of the household	Poor	581	42.47
	Middle-class	273	19.96
	Rich	514	37.57
Media exposure	No	662	48.39
	Yes	706	51.61

Other^*a^ = single, divorced, and widowed; other^*b^ = private employee, NGO employee and daily laborer.

### Risk factors for community-acquired pneumonia among under-five children

In the bivariable logistic regression analysis, place of residence, education status of the mother, occupational status of mother, household wealth index, age of the child, family size, current age of mother, media exposure, religion, vaccination status, duration of breast feeding status, age at complementary feeding, having diarrhea in the last 2 weeks, history of lower respiratory tract infection in the last 2 weeks, history of upper respiratory tract infection in the last 2 weeks, place of delivery, source of drinking water for HH, child stays during cooking, and presence of separate kitchen for household were significantly associated with community-acquired pneumonia at a *p*-value of <0.25 and entered into the multivariable logistic regression model.

In the multivariable logistic regression model, educational status of mother/caregiver/, stunting, religion of mother, history of having diarrhea in the last 2 weeks, history of lower respiratory tract infection in the last 2 weeks, history of upper respiratory tract infection in the last 2 weeks, and presence of separate kitchen for household had a statistically significant association with CAP at the 5% significance level.

This study showed that children who had a history of acute lower respiratory tract infection in the last 2 weeks were 15 times [AOR = 14.57; 95%CI 3.18, 66.78] more likely to develop community-acquired pneumonia as compared to their counterparts. Similarly, children from households with a history of acute upper respiratory tract infection within the last 2 weeks prior to data collection were nine times [AOR = 9.06; 95%CI 2.03, 4,042] more likely to develop community-acquired pneumonia as compared to their counterparts.

Children who had a history of diarrhea in the past 2 weeks were four times (AOR = 3.75; 95% CI: 1.96, 7.18) more likely to develop CAP compared to their counterparts.

The estimated odds of having community-acquired pneumonia in children whose mother attained primary education were 0.38 times less likely compared to children whose mother did not attain education (AOR = 0.38; 95%CI: 0.15, 0.95). Similarly, the odds of having community-acquired pneumonia in children were five times (AOR = 4.80; 95% CI: 2.31, 9.94) more likely among stunted children than among normal children.

This study revealed that children from those households that had a separate kitchen for cooking purposes were 0.38 times decreased in the risk of community-acquired pneumonia compared to children from those households that had no separate kitchen from the main house (AOR = 0.38; 95%CI: 0.20, 0.72) (Table 6).

**Table 6:** Bivariable and multivariable logistic regression analyses of community-acquired pneumonia and its associated risk factors among under-five children in the Awi Zone, Northwest Ethiopia (*n* = 1,368).

Characteristics	Categories	COR (95% CI)	AOR (95% CI)	*p*-value
Age of the child in months	Less than 6	1	1	
	6–11	2.63 (0.97,7.18)*	0.87 (0.02,31.34)	0.937
	12–23	5.11 (2.01,12.99)*	13.90 (0.36,542.75)	0.159
	24–59	2.32 (0.91,5.91)*	3.50 (0.09,143.05)	0.509
Child’s birth order	First	1		
	2–3	0.92 (0.59,1.42)		
	4–5	1.06 (0.66,1.69)		
	6 and above	0.85 (0.49,1.49)		
Number of children in the family	1	1		
	2 and above	1.05 (0.71,1.54)		
Family size	Less than 5	1	1	
	5 and above	1.64 (1.16,2.34)*	1.45 (0.74,2.8300)	0.275
Educational status of mother/caregiver/	No education	1	1	
	Primary	0.67 (0.44,1.04)*	0.38 (0.15,0.95)	0.038**
	Secondary and higher	0.94 (0.62,1.42)	1.39 (0.543.58)	0.492
Marital status	Married	1		
	Other^*a^	0.61 (0.24,1.54)		
Media exposure	No	1	1	
	Yes	0.70 (0.50,0.99)*	0.90 (0.42,1.92)	0.782
Main source of drinking water	Unprotected	1	1	
	Protected	0.64 (0.42,0.96)*	0.86 (0.39,1.91)	0.707
Toilet facility	No	1		
	Yes	0.84 (0.46,1.55)		
Wealth index of the family	Poor	1	1	
	Middle-class	0.68 (0.43,1.06)	0.83 (0.35,1.97)	0.669
	Rich	0.54 (0.37,0.80)*	0.76 (0.28,2.09)	0.593
Residence	Urban	1		
	Rural	1.03 (0.73,1.47)		
Sex of child	Male	1		
	Female	1.14 (0.81,1.59)		
Religion	Orthodox	1	1	
	Other	0.29 (0.09,0.92)*	0.07 (0.02,0.32)	0.001**
Nutritional status of the child height for age (41)	Normal	1	1	
	Stunting	1.52 (1.06,2.18)*	4.80 (2.31,9.94)	<0.001**
Place of delivery	Home	1	1	
	Health facility	0.56 (0.32,0.99)*	2.85 (0.84,9.65)	0.093
Child initiated complementary feeding at 6 months	No	1	1	
	Yes	0.73 (0.52,1.03)*	0.83 (0.46,1.47)	0.517
	No starting	0.28 (0.11,0.71)*	0.65 (0.02,24.91)	0.819
Child vaccination	Partially	1	1	
	Fully	0.69 (0.47,1.01)*	0.72 (0.32,1.58)	0.409
Having diarrhea in the last 2 weeks	No	1	1	
	Yes	5.82 (4.07,8.31)*	3.75 (1.96,7.18)	<0.001**
History of acute lower respiratory tract infection in the last 2 weeks	No	1	1	
	Yes	102.62 (62.20,169.30)*	14.57 (3.18,66.78)	0.001**
History of acute upper respiratory tract infection in the last 2 weeks	No	1	1	
	Yes	98.61 (60.37,161.07)*	9.06 (2.03,40.42)	0.004**
Cooking practices in the family	Electricity	1		
	Charcoal	1.19 (0.15,9.44)		
	Wood	2.48 (0.33,18.79)		
Child stays during cooking	Out of the cooking room	1	1	
	On the mother’s back	1.45 (1.00,2.11)*	1.79 (0.94,3.42)	0.076
Presence of a separate kitchen for the household	No		1	
	Yes	0.45 (0.32,0.63)*	0.38 (0.20,0.72)	0.003**

*Significance at a *p*-value of <0.25; **Significance at a *p*-value of <0.05; AOR and *p*-value cells are blank because the COR values were insignificant.

## Discussion

The finding of this study revealed that the 2-week percentage of community-acquired pneumonia among under-five children of age was 11.33% (95% CI: 9.75–13.12%). This finding is compatible with the study conducted in Northwest Ethiopia (12%) (28). This consistency might be due to the launching of the Health Extension Program (HEP), improving access to health care to meet the primary attention of the MDG agenda and the introduction of the integrated community cause management program (29). The percentage of community-acquired pneumonia in this study area is lower than studies conducted in the University of Gondar Referral Hospital, Ethiopia (18.5%) (30), Munesa District, Arsi Zone, Oromia Region, Ethiopia (17.7%) (23), Gamo Zone, Southern Ethiopia (30%) (31), and East Africa (34%) (8). The difference might be attributed to the variation in the socio-demographic, seasonal, behavioral, and environmental factors of study households compared to other studies, such as people’s way of life, educational levels of the community, maternity care of child’s immunization, nutritional interventions, and community-based health education programs. However, it is higher than the national prevalence of pneumonia at 7% (32), Angolela Tera district, North Showa, Ethiopia (5.8%) (20), Debre Birhan (5.5%) (33), and Mali (6.7%) (34). This discrepancy might be due to differences in sample size, sampling method, skills of data collectors, and methodology.

The findings of this study revealed that educational status of mother/caregiver/, stunting, religion of mother, history of having diarrhea in the last 2 weeks, history of lower respiratory tract infection in the last 2 weeks, history of upper respiratory tract infection in the last 2 weeks, and presence of separate kitchens for household were identified to be significant factors associated with the occurrence of community-acquired pneumonia among under-five children.

AURTI showed a statistically significant association with the occurrence of CAP, in which the odds of developing CAP among children living in households that had a history of AURTI in the last 2 weeks were nine times more likely to develop CAP compared to children living in households that had not had AURTI. This finding was supported by previous studies conducted in the Oromia zone, Ethiopia (15), in Kersa District, Southwest Ethiopia (35), in Kenya (17), and in other East African countries (8). This can be explained by the fact that upper respiratory tract infections weaken child feeding habits by making them anorexic. This lowers the children’s nutritional status and weakens their immune systems, which increases the risk of community-acquired pneumonia (35).

Children who had a history of diarrhea in the past 2 weeks prior to data collection were four times more likely to develop community-acquired pneumonia compared to their counterparts. This finding was in agreement with the previous study conducted in Tigray, Ethiopia (36), in urban areas of Oromia special zone of the Amhara region (15). This can be explained by the fact that children who have a concomitant illness such as diarrhea may have a lowered immune system, making them more susceptible to diseases such as pneumonia (12).

According to this study, children who had a history of ALRTIs in household members in the past 2 weeks prior to the data collection were 15 times higher risk of having CAP. It is consistent with an institutional-based study conducted in Kemise, Oromia zone, Amhara Region (26). This finding could be due to lower respiratory tract infections that were contagious and are easily transmittable from household contacts to children. These infections were often viral in origin and may be viewed as the consequence of progression from milder forms of lower respiratory tract predispose children to pneumonia. The severity of the disease also depends on the virulence and load of the pathogen; the load is usually higher when the infection is from a household contact (37). Moreover, stunting was also identified as a risk factor for community-acquired pneumonia in this study. Children who are stunted have a five times higher prevalence of getting community-acquired pneumonia as compared to normal children. This finding was consistent with studies conducted in Hosanna, Hadiya Zone, Ethiopia (12) and Bangladesh (38). The possible explanation for this association could be based on the fact that stunting indicates long-term malnutrition of the children, which weakens the child’s natural body defense mechanism, and the child becomes susceptible to the infection-causing agent, making the child vulnerable to pneumonia. From different perspectives of different studies, malnutrition weakens the respiratory muscles needed to clear secretions in the respiratory tract, which, in turn, predisposes one to pneumonia (39).

According to this study, the presence of a separate kitchen from the main house for cooking was significantly associated with community-acquired pneumonia among under-five children. The odds of community-acquired pneumonia among under-five children who were from households having separate kitchens from the main house for cooking were 0.38 times less likely compared to their counterparts. This finding is consistent with a study conducted in the Wondo Genet district in the Sidama Zone, Ethiopia (14). This could be the reason that the risk of household air pollution is high, which contributes to pulmonary inflammation and tissue damage that favors the growth of ethologic agents and increases the susceptibility of children to acquire pneumonia.

This study showed that the educational level of a mother had a significant effect on community-acquired pneumonia. This result is supported by a study in the slums of Dibrugarh town (40). Educated mothers recognize the signs and symptoms of pneumonia early and easily understand community-based interventions, efforts to improve nutrition, and improved access to healthcare services. Thus, their children have a better outcome than others.

### Strength and limitation of the study

The strength of this study was that the analysis used primary data and ensured the quality of data with the standardized data collection tool, and a pretest was conducted before the actual data collection. As a limitation, the study was based on a cross-sectional study design, which may not set a temporal relationship between cause and effect. The diagnosis of pneumonia was based on clinical WHO IMNCI classification guidelines, which could introduce misclassification bias. There might be a possibility of recall, limit social desirability, and interviewer bias due to the retrospective tracking of information that will result in underreporting and misreporting of events. However, attention was given to the study procedures, including the process of training data collectors and close supervision throughout the activity to minimize expected biases. Since the study was conducted at a community-based based, blood tests with pulse oximetry and chest X-ray tests were not used. However, failure to use this method in the present study may underestimate the prevalence of pneumonia among the study subjects. Some variables such as radiological findings, anemia, antibiotics, and hospital admission data, were not included. Hence, we recommend that future researchers include those variables.

## Conclusion

This study revealed a relatively high percentage of community-acquired pneumonia among under-five children in the study area. The educational status of mother/caregiver/, stunting, religion of mother, history of having diarrhea in the last 2 weeks, history of acute lower respiratory tract infection in the last 2 weeks, history of acute upper respiratory tract infection in the last 2 weeks, presence of separate kitchen for household were significantly associated with community-acquired pneumonia among under-five children in the Awi Zone, northwest Ethiopia. Therefore, we recommend appropriate and adequate health education regarding nutritional intervention, prevention and early treatment of diarrhea and ARTI, and prevention of indoor air pollution to reduce the risk of community-acquired pneumonia.

## Data Availability

The raw data supporting the conclusions of this article will be made available by the authors, without undue reservation.
